# Brazilian breast cancer patient-reported outcomes: What really matters for these women

**DOI:** 10.3389/fmedt.2022.809222

**Published:** 2022-11-21

**Authors:** Aline Silveira Silva, Ana Cláudia Wekmuller França, Matheus Piccin Padilla, Luana Schroeder Macedo, Carlos Alberto da Silva Magliano, Marisa da Silva Santos

**Affiliations:** ^1^ Grupo de Pesquisa em Acesso a Medicamentos e Uso Responsável, University of Brasilia (AMUR-UNB), Brasilia, DF, Brazil; ^2^ Núcleo de Avaliação de Tecnologias em Saúde, National Institute of Cardiology (NATS-INC), Rio de Janeiro, RJ, Brazil; ^3^ The Federal University of the State of Rio de Janeiro, Rio de Janeiro, RJ, Brazil

**Keywords:** patient-reported outcomes, PRO, PROs, patient preferences, preference study, breast cancer, patient perspective, patient experience

## Abstract

**Introduction:**

Patient-Reported Outcomes (PRO) are directly reported by the patient without interpretation of the patient's response by a clinician or anyone else and pertains to the patient's health, quality of life, or functional status associated with health care or treatment. It can provide patients’ perspectives regarding treatment benefit and harm beyond survival and are often the outcomes of most importance to patients. This study aims to describe and analyze outcomes reported by Brazilian women diagnosed with breast cancer and rank the most important attributes for these patients.

**Methods:**

Observational descriptive study composed of exploratory interviews followed by online questionnaires applied to a convenience sample of women diagnosed with breast cancer.

**Results:**

Twelve women were interviewed to explore the main outcomes and preferences about their treatments, such as the most common side effects and the most impacted aspects of life after diagnosis and BC treatment. Psychological, emotional, and sexual impacts were frequently described as impacted aspects. Fifty-three women, from all the five Brazilian regions, answered the online questionnaire. Following an order of importance ranking, the following outcomes were chosen, respectively: overall survival, progression-free survival; and quality of life. The treatment effects that were considered less important, among this sample, were pain and adverse events.

**Conclusions:**

Thinking about expanding the therapeutic quality of users, it is essential to take into account the experiences of patients. PRO is a trend in current research to achieve this goal, in order to influence the decisions of HTA agencies about the importance of valuing outcomes that affect patients' lives.

## Introduction

Breast cancer has been a major public health problem. It is the second most incident cancer in the world and the most prevalent in women, besides being the second worldwide leading cause of cancer mortality (1).

Data from 1980 to 2006 showed that breast cancer mortality has been increased in all five major geographic regions of Brazil (2). Only in 2019, the Brazilian Mortality Information System recorded 18,296 deaths in women due to breast cancer, the principal cause of death from cancer in Brazilian women. Estimates for each 2020–2022 period indicate that there will be about 66 thousand new cases in the country. It is noteworthy that mortality rates are strongly related to access to health services and the quality of care that is offered to women (3).

As therapeutic options for breast cancer, primary tumor surgery, assessment of axillary involvement and radiotherapy as a form of local treatment and systemic drug treatment, which consists of chemotherapy and hormone therapy (4) In cases of resistance, new therapeutic options are used. One of these options is a combination of CDK4/6 inhibitors in combination with hormone therapy, which has been shown to be effective in women with advanced breast cancer negative for human epidermal growth factor receptor 2 (HER2), positive hormone receptor (HR+) (5).

In the Brazilian Public Health System, the current clinical guidelines recommend only hormone therapy as the first-line therapy for postmenopausal women with advanced luminal BC (6). However, international guidelines recommend adding CDK 4/6 inhibitors (such as Abemaciclib, Palbociclib, or Ribociclib) in the first-line therapy (7), since an increase in overall survival (OS) and progression-free survival (PFS) have been demonstrated in pivotal studies using these drugs in advanced luminal breast cancer (BC) (8–14).

OS has long been the gold standard outcome in establishing the efficacy of oncology therapies and PFS, defined in clinical trials as the time from randomization until first evidence of tumor progression or death from any cause, is commonly used as a surrogate endpoint, which has been questioned by some cancer researchers, often without an evaluation of patient preferences (15).

The patient experience has played an increasingly important role in clinical research since it is now understood that a whole system, such as a patient-centered approach, is required for a thorough assessment of the impact of therapy and care (16). In the last decade, the focus on the patient has become a key concept in research (17) and several health technology assessment (HTA) agencies promote patient engagement in the decision-making process as well (18).

Patient-Reported Outcomes (PRO) are directly reported by the patient without interpretation of the patient's response by a clinician or anyone else and pertains to the patient's health, quality of life, or functional status associated with health care or treatment (19). PRO instruments can provide patients' perspectives regarding treatment benefit and harm, directly measure treatment benefit and harm beyond survival, and are often the outcomes of most importance to patients. PROs can be used either as a secondary outcome of a study, to complement primary outcomes, such as survival rates, or as a primary outcome, when there is no objective out­come measurement (16).

According to an investigation about how inclusion of PRO evidence has evolved and influenced recommendations by HTA agencies (G-BA, HAS, NICE and SMC), 72% of the drug indication combinations included PRO data in one or more submissions. It shows that, however it is not yet a standard practice, HTA agencies tend to value the submission of PRO data and it can have a positive influence on recommendations (20).

In Brazil, Progress-free survival (PFS) is considered a substitute outcome in the guidelines for the treatment of breast carcinoma, which means, a PFS is not considered an important factor in this decisive process of incorporating a drug as an option in the Brazilian public health system (SUS) (6). However, based on consideration of patient preferences in the decision-making process, agencies such as the Canadian Agency for Drugs and Technologies in Health (CADTH), National Institute for Clinical Excellence (NICE), and the Scottish Medicine Consortium (SMC) have indicated that PFS is an important outcome for breast cancer patients, as it allows them to maintain their usual activities for a longer period. In addition, it was identified that patients would be willing to accept adverse events resulting from endocrine therapy so that they could postpone the need for chemotherapy, which is associated with higher toxicity and decreased quality of life than endocrine therapy. These HTA agencies seem to consider PFS in decision making, since all of them have approved drugs associated with PFS gain, such as CDK inhibitors.

In this way, the influence of patient participation in the decisions of HTA agencies is evident. Therefore, the present study aims to identify the main relevant outcomes for patients with breast cancer in Brazil, as well as to describe and rank important attributes and outcomes for these patients.

## Materials and methods

This is an observational study composed of exploratory interviews followed by online questionnaires applied to a convenience sample of women diagnosed with breast cancer. The recruitment was carried out virtually between June and October 2020, through an online form, released by the research team in partnership with patients support Non-Governmental Organizations (NGOs), which are “*Oncoguia*”, “*Recomeçar*”, “*Zen Cancer*” and “*Colabore com o futuro”*. The recruitment form received 46 responses. The following inclusion criteria was established: Brazilian women diagnosed with breast cancer who already have been or were being treated for breast cancer. After this initial recruitment, we firstly invited 29 women that answered the recruitment form and completed the inclusion criteria to be part of this study.

Data collection occurred first through individual exploratory interviews conducted by a trained researcher over the phone and lasted for approximately 30 min. The aim of the interviews was to capture important outcomes, preferences, and other results from patients, with the potential to provide insights that could help to answer the questions of this research. Twelve women were interviewed using a convenience strategy determined by theoretical saturation of the discourses related to the outcomes of the disease and treatment.

Subsequently, an online questionnaire was applied to the interviewed women and other patients that preferred to participate only in this phase of the study. The questionnaires were sent to the same patients who participated in the interviews and the NGOs cited above also helped to spread the survey among other breast cancer patients. The aim of this second phase was to classify and rank the attributes and outcomes previously identified in the interviews, in addition, to explore more the ethical, social, and patient aspects of this disease and its treatment from the perspective of the Brazilian breast cancer patients'.

The semi-structured interview questionnaire (Questionnaire 1) was developed considering the literature on the topic and the expertise of specialists, with the objective of collecting reports of patients' experiences and preferences, about important attributes and the classification of these. The online questionnaire (Questionnaire 2) was elaborated with close-ended questions using multiple-choice questions to measure Quality of Life and a Likert scale to rank the attributes and outcomes found in the conducted interviews.

All data were collected between September and November 2020. All participants provided informed consent prior to their participation.

To analyze the speeches of the interviews, a verbatim transcript was carried out in full, and the data of the interviewees were anonymized, using only the initials and thematic analysis (21). The thematic analysis is a qualitative analysis technique characterized by flexibility, as it is essentially independent of a specific theory or epistemology and can be applied with a variety of theoretical and epistemological approaches. The content analysis was peer review by two researchers of the team.

## Results

Twelve women were interviewed in September 2020. They were from different Brazilian cities in the Southeast Region (São Paulo, SP; Rio de Janeiro, RJ; and Minas Gerais, MG), and in the Mid-West Region (Distrito Federal, DF). Most patients were 50–69 years old, and only three were between 30 and 49 years old. Among the interviewed patients, only two of them were diagnosed with metastatic breast cancer. Only two of the interviewed patients were diagnosed with metastatic breast cancer. Fifty-three women from all five Brazilian regions answered the online questionnaire applied in October and November 2020. These breast cancer patients were between 30 and 69 years old and eleven were diagnosed with metastatic breast cancer. Most participants were diagnosed with BC diagnosis between 2018 and 2020, i.e., in the last two years (Table 1).

**Table 1:** Characteristics of the participants.

		Number of participants % (*n*)	
		Interview (*n* = 12)	Survey (*n* = 53)
**Age**			
	<30	0	0
	30–49	25 (3)	49 (26)
	50–69	75 (9)	47 (25)
	>70	0	0
	NI	0	4 (2)
**City/State**			
Southeast Region	Rio de Janeiro, RJ	60 (7)	24 (13)
	Duque de Caxias, RJ	8 (1)	0
	São Paulo, SP	8 (1)	5 (9)
	Contagem, MG	8 (1)	0
	Aparecida, SP	0	2 (1)
	Hortolândia, SP	0	2 (1)
	Itu, SP	0	2 (1)
	Praia Grande, SP	0	2 (1)
	Santo André, SP	0	2 (1)
	Santos, SP	0	6 (3)
	Belo Horizonte, MG	8 (1)	4 (2)
Mid-West Region	Brasília, DF	8 (1)	15 (8)
South Region	Morretes, PR	0	2 (2)
	Florianópolis, SC	0	2 (1)
North Region	Belém, PA	0	4 (2)
Northeast Region	Fortaleza, CE	0	22 (12)
	Caucaia, CE	0	2 (1)
**Diagnostic**			
	BC	83 (10)	79 (42)
	Metastatic BC	17 (2)	21 (11)
**Current drug treatment**			
	Tamoxifen	67 (8)	30 (19)
	Anastrozole	17 (2)	13 (8)
	Letrozole	17 (2)	8 (5)
	Pembrulizumab	0	0.5 (1)
	Pabociclib	8 (1)	0.5 (1)
	Pertuzumab	8 (1)	3 (2)
	Trastuzumab	8 (1)	5 (3)
	Goserelin acetate	17 (2)	5 (3)
	Zoledronic acid	0	5 (3)
	Chemotherapy	0	3 (2)
	Exemestane	0	5 (3)
	Does not take any drug	17 (2)	22 (14)
**Time since first BC diagnosis**			
	< 2 years (2018–2020)	NA	45 (24)
	2–4 years (2016–2017)	NA	28 (15)
	4–6 years (2014–2015)	NA	6 (3)
	6–8 years (2013–2012)	NA	9 (5)
	>8 years (<2011)	NA	8 (4)
	NI	NA	4 (2)

BC, breast cancer; NA, not applicable; NI, not informed; RJ, Rio de Janeiro; SP, São Paulo; MG, Minas Gerais; DF, Distrito Federal; PR, Paraná; SC, Santa Catarina; PA, Pará; CE, Ceará.

### Interviews

Tamoxifen was the most widely used breast cancer medication among study participants (*n* = 8). One of the patients reported that, due to side effects, she recently had to stop taking anastrozole, and is currently taking only tamoxifen (Table 1).

**Figure 1:**
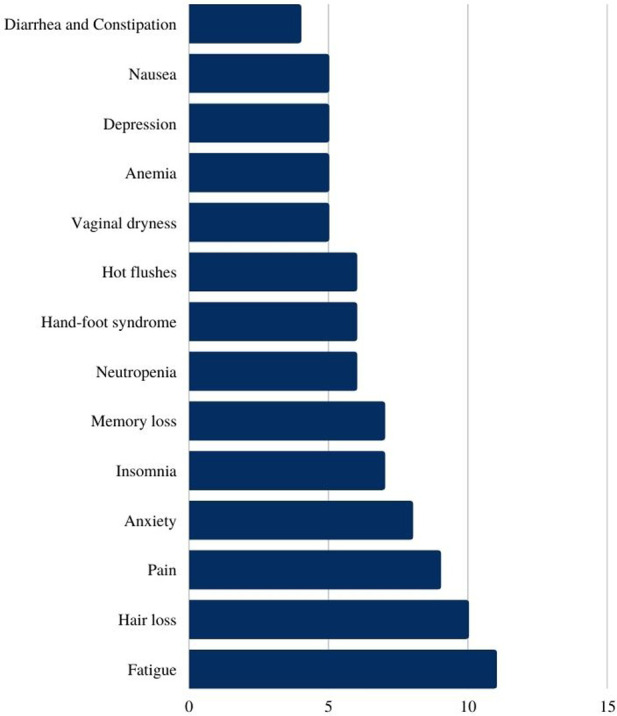
Most reported side effects related to breast cancer treatment (absolute numbers).

The most reported side effect related to breast cancer treatment was fatigue (*n* = 11) and hair loss (*n* = 10) (Figure 1). Other side effects such as loss of appetite, heartburn, dyspnea, osteopenia, difficulty concentrating, general dryness and peripheral neuropathy were also reported during the interview.

**Psychological and emotional** impact were frequently described, even though we didn't specifically mention these aspects in the interview (we’ve given as examples physical, sexual, social, and economic aspects). Some of the patients used similar sentences to narrate the difficult moment of the diagnosis, as seen below.

“It was such a surprise because I used to take care of myself, I always had healthy habits” (MCTR, 56 years old, BC)

“It was a huge surprise… I didn’t have any node, nothing. It was discovered during the annual checkup and I was in shock. Despite we know there's a treatment, you feel too afraid of the future (TS, 56 years old, BC)

“My life was totally affected. My life was very different before cancer. When I was diagnosed, I got deeply depressed, I was sure I was going to die. It was an enormous suffering” (TMLPRA, 61 years old, BC)

“I feel much more fragile after the diagnosis and with this treatment, I cry frequently… I feel like a baby” (PEBB, 45 years old, metastatic BC)

Mental health was described as something important during the treatment and it seems integrative practices - such as natural foods, meditation, yoga - have importance on this aspect for some of the interviewed oncological patients, as we can see in the following quotes:

“I found the Zen Cancer Institute and I could stop 1 year to take care of myself, look at my mental health, self-knowledge, meditate, integrative practices like yoga. I allowed myself to do things that are good for me, that brings me positivity. That was the greatest positive impact: a better mental health” (RMBL, 44 years old, BC)

“I”m pretty sure 50% of my results were due to the integrative medicine associated with the traditional treatment” (LVSG, 61 years old, BC)

Impact on self-esteem was also reported and it was frequently related to the loss of hair and to the mastectomy. Many patients continue to undergo psychotherapeutic follow-up since the breast cancer diagnostic.

“I never worried about appearance and then, I started to change it, to go out on the streets and even at home with my family. My husband”s support was fundamental for my self-esteem maintenance” (MCTR, 56 years old, BC)

“Cancer affected everything, in terms of life expectance, quality of life, sexually speaking, my self-esteem… I had depression and I”m still treating it with a psychiatrist” (DSG, 44 years old, BC)

When asked about what aspects of life were more affected, all patients mentioned that the disease had a strong physical impact, mainly related to consequences of the chemotherapy and surgery (mastectomy and axillary lymph node dissection). Pain, limiting fatigue, appearance changes – due to loss of weight, hair and breasts, loss of strength, and balance limiting exercise routine were some of the described physical impacts.

“The physical aspect, because I didn”t do the breast reconstruction surgery, so to look at me in the mirror, my sexuality, my relationship with my husband are aspects that are getting better little by letter. Tamoxifen induced my menopause. I still can”t do exercise; I don”t have good mobility on my left arm because they extracted 4 lymph nodes” (DSG, 44 years old, BC)

“I wake up feeling pain, I have gastric reflux and other gastric adverse effects because of the many medicines I take” (PEBB, 45 years old, metastatic BC)

Six patients related negative impacts on sexual life. Vaginal dryness and loss of libido were frequently reported as the cause but a strike on self-esteem and emotions was also reported as possible reasons to affect this aspect.

“My libido decreased a lot during chemotherapy. But my husband respected this moment, supported and understood me. It was getting better after the chemo and surgery” (MRAS, 56 years old, BC)

“It”s so complicated, depressing. There is vaginal dryness, atrophy, there is no libido, the act hurts, it becomes mechanical, it is no longer pleasant. It was the most affected part” (PEBB, 45 years old, metastatic BC)

According to most of the interviewed patients, the impact on social life was surprisingly positive. Many women reported that, despite they have found some stigma coming from society, they had an improvement in social life, mainly when there was a strong support network like family, close friends and Cancer Support Non-Governmental Organizations. However, some patients described uncomfortable feelings due to the lack of knowledge about the disease. On one hand, some people approached them like cancer was a death sentence or, on the other hand, others didn't understand how the patient was sick if they were apparently doing so great. The importance of cancer awareness, besides better access, was cited as an important action that should be established by health authorities.

“Social aspects were positively affected because to deal with other people got easier for me. Since I had to go through all this suffering, I am more empathetic. Things that used to annoy me, don”t bother me anymore. I feel more like a conciliator between people nowadays” (AFS, 52 years old, BC)

“Socially, I felt a lot of support from friends, I was not isolated nor marginalized, I felt welcomed. There were some restrictions, the surgery was in the summer, so I became more secluded, quieter, but it is not difficult to live with herself (…) There were people visiting. At work, I didn't feel any prejudice. Although there is still a stigma, people are terrified, scared. It was difficult to deal with a diagnosis that nobody wants to have” (ACF, 53 years old, BC)

“The social aspects were very affected. With close people, I didn”t have any problem, I have friends and family supporting me. But I had difficulty with other people, mainly after I cut my hair before losing it completely, they noticed I had something. Most people didn”t know at work, so it was hard to deal with it” (MCTR, 56 years old, BC)

“Someone asked, “You have cancer, are you going to die?” and I replied, “I'm going, aren't you?” Cancer is not punishment, I do not see it as punishment, but as a school, a great learning experience, I am a better person than I was before.” (JG, 58 years old, Metastatic BC)

Half of the interviewed patients (*n* = 6) reported breast cancer brought an economic impact on their lives, mainly for patients that rely on public health system or those who were unemployed or had to stop working because of the disease or its treatment. But even patients who have health insurance reported impact due to the need for expending more money on healthier food, medicines, exams, and doctors which were not covered. All patients that mentioned not having an economic impact had health insurance.

“Economically affected me a lot, mainly in the beginning, because I had to get a medical leave and my salary was lower and I had a lot of expenses with medicines that my health insurance didn”t cover. But after I retired from one of my jobs, things got better” (AFS, 52 years old, BC)

“I used to work but now I”m retired because of the disease. I spent a lot of money on physiotherapy and medicines. I do not have health insurance, my treatment is under SUS, but some medicines are not available, they are costly, and we have to buy it” (DSG, 44 years old, BC)

The exam routine was another frequently mentioned stress factor. The fear of another diagnosis was present in most analyzed discourses.

“When we have a diagnostic like this, you live with fear all the time, because it”s something that you would never imagine happening in your life… it comes from nowhere and it can always come back” (TMLPRA, 61 years old, BC)

“Every year I do mammography and I get so scared; I don”t want to go through everything again. We”re always scared, every year I go to do mammography I get scared and nervous” (RP, 68 years old, BC)

“I”m terrified of having a recurrence. I have depression and anxiety, so something that affects me a lot is this fear of having cancer again” (LVSG, 61 years old, BC)

### Online questionnaire

**Figure 2:**
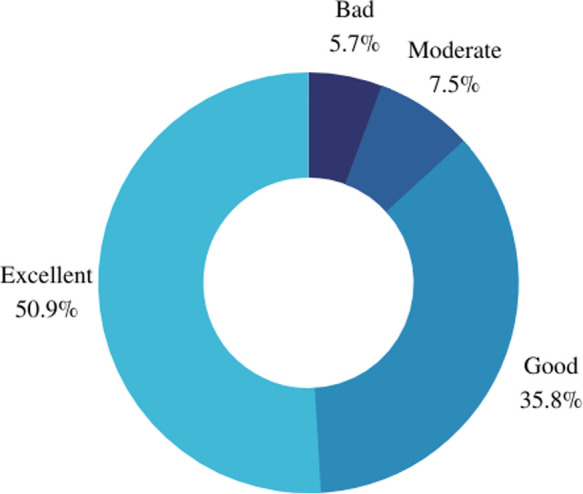
Self-declared health state in the last seven days.

**Figure 3:**
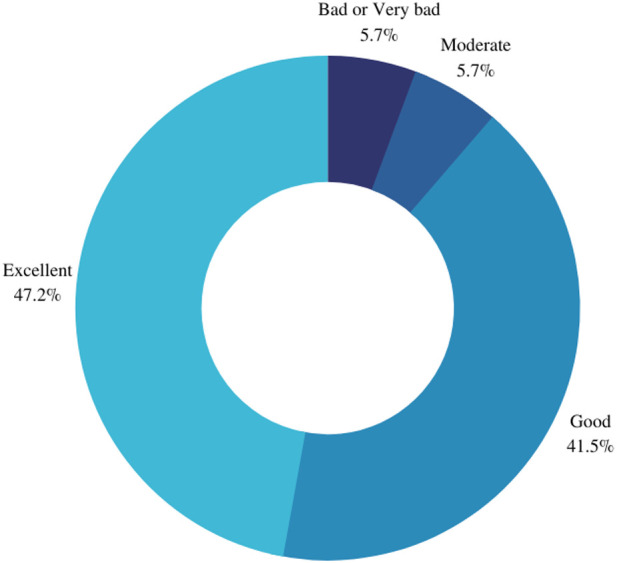
Self-declared quality of life in the last seven days.

When inquired to report how they would evaluate their health state in the last seven days most participants declared “excellent” health state (*n* = 27, 51%), followed by “good” (*n* = 19, 36%), “moderate” (*n* = 4, 7%) and “bad” (*n* = 3, 6%) (Figure 2). Similar results were observed when the patients were inquired to report how would they evaluate their quality of life in the last seven days. Most women self-reported “excellent” quality of life (*n* = 25), followed by “good” (*n* = 22), “moderate” (*n* = 3), and “bad” (*n* = 2) and “very bad” (*n* = 1) (Figure 3).

**Figure 4:**
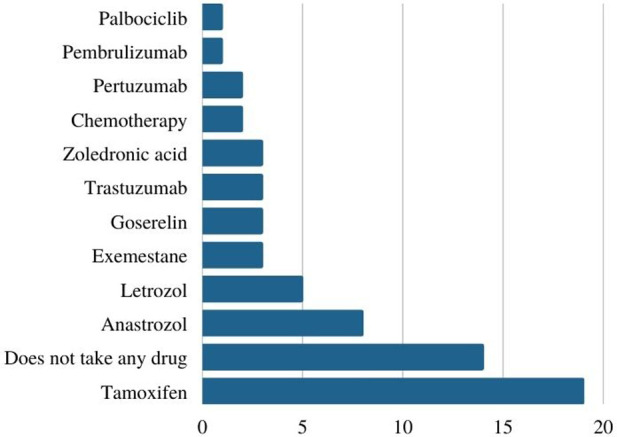
Current breast cancer drug treatment (absolute numbers).

When asked about their current drug treatment, seven patients declared having two or more associated oral medicines as part of their current treatment. Most of the patients that answered this survey (*n* = 19) reported tamoxifen as part of their current oral therapy, followed by patients who were not taking any medicine (*n* = 14) and patients taking anastrozole (*n* = 8) — in association with other drugs or not. Both patients that reported being under chemotherapy were diagnosed with metastatic breast cancer at the time of this survey (Figure 4). As for the duration of use of the drugs mentioned above, twenty-five women reported their use for more than ten months.

**Figure 5:**
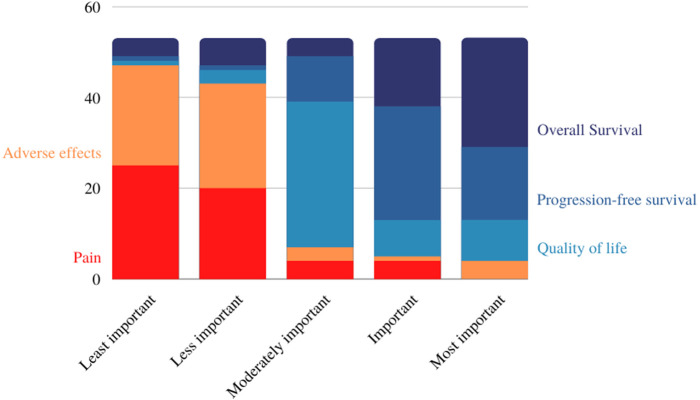
Treatment outcome ranking (absolute numbers).

The treatment effects that were less and the least important were adverse events (*n* = 23) and pain (*n* = 25), respectively (Figure 5). Here, it's important to consider the main profile of these patients, mostly breast cancer patients without metastatic disease. The increase of the overall survival was considered the most important (*n* = 24) and progression-free survival was considered an important effect (*n* = 25).

**Figure 6:**
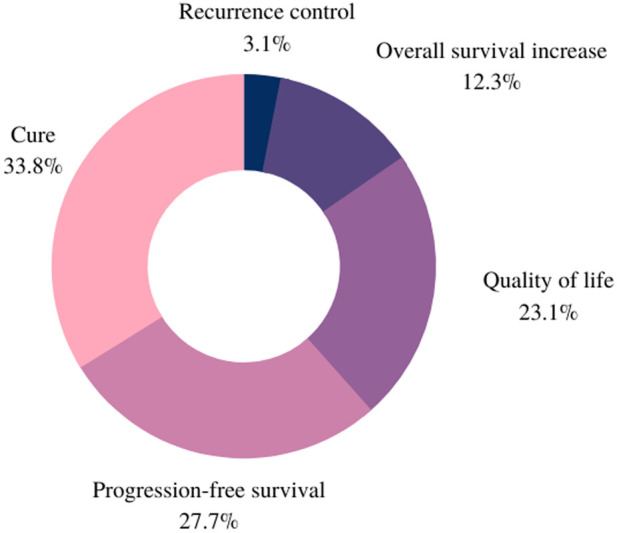
The main treatment objective.

When asked “what is your main treatment objective?”, most women considered cure (*n* = 39), progression-free survival (*n* = 7) and quality of life (*n* = 5) as the main goals of their treatment - even if they were diagnosed with metastatic disease. When faced with the possibility of cure, overall survival increase (*n* = 1) ranked behind quality of life. One patient who was under chemotherapy treatment mentioned “to reduce tumor size” as her main treatment objective (Figure 6).

**Figure 7:**
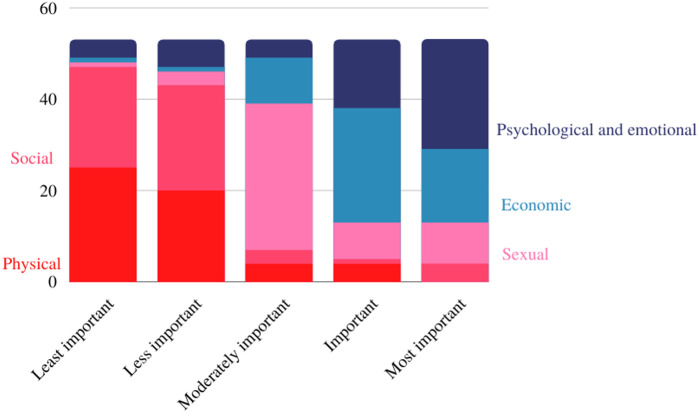
The most impacted aspects of life.

The replies to the surveys revealed that, when asked to choose the aspects of life that breast cancer had the greatest impact on, the psychological and emotional component represented the most important (*n* = 29). The economic aspect was considered important (*n* = 15) by the Brazilian patients, followed by the sexual aspect, considered moderately important (*n* = 15). Social aspect was considered less important (*n* = 19) and physical aspect was evaluated as the least important (*n* = 18) (Figure 7).

## Discussion

As far as we know, this is the first study that sought to understand the most important outcomes for breast cancer patients in Brazil. Typically, studies focus almost exclusively on clinical outcomes, that is, they focus only on what researchers consider important. In this study, we found that clinical outcomes such as cure, overall survival, survival free of progression (which Brazilian patients call “controle da doença”, translated as progression-free survival), and quality of life are important for Brazilian patients, but so are other secondary outcomes that showed large impact on these women's lives. For example, for patients who responded to the online questionnaire, the condition itself and its treatment have a great psychological and emotional impact, an aspect considered as the most important when compared to physical outcomes or social, sexual and economic impact. The sexual aspect was found as moderately important, and is related to frequently reported adverse effects, such as decreased self-esteem and libido, vaginal dryness, but may also be related to the emotional impact of the disease on these patients lives.

According to our findings, overall survival and progression-free survival are the most important treatment outcomes, showing that our findings are aligned with a recent survey that sought to rank the most valued outcomes for cancer patients (22). Despite this, some authors question the relevance of this outcome. However, recent research revealed that patients who remain in the PFS state might postpone chemotherapy. Also, using of PFS results is essential to support other outcomes in economic analyses regarding breast cancer treatment (23).

It is noteworthy to highlight that the women interviewed frequently reported the condition and its treatment as having a significant physical impact. Adverse events such as pain and fatigue have often been described. However, when analyzing the sample of women who answered the online questionnaire, the physical aspect was described as the least important, when compared to other aspects. For these patients, cure or an overall survival were the most expected treatment results, followed by progression-free survival and quality of life. The treatment effects considered less important were adverse events and pain was showed as the least important effect. Here, it is important to consider the main profile of this sample, formed mainly by patients with breast cancer without metastatic disease. Of a total of 65 women who participated in this study (through both interviews and online questionnaires), only thirteen were diagnosed with metastatic cancer.

A limitation of this study is the small sample of participants, mainly when compared to the total population of Brazilian women with breast cancer. The estimation for the triennium of 2020 to 2022 was 662,80 new cases in Brazil (24). Therefore, we understand this is not a representative sample. Another limitation is the fact that the data collection instrument used was not a validated questionnaire. Due to the short time, the research team decided to apply a questionnaire that could be feasible, in addition to reflecting the perspective of Brazilian women regarding the disease and its treatment in relation to the ethical, social impacts, and other results reported by the patient. It is noticed that the instrument built and used could capture some issues that generic instruments cannot, due to the lack of sensitivity. Some of the important aspects and results captured by this research are not addressed in most generic instruments, as is the case with SF-6 or EQ-5D.

In this sense, the importance that health-related quality of life has for women diagnosed with breast carcinoma is evident. However, this outcome is often overlooked in clinical studies of different cancer treatment options, in which priority is given to overall survival, for example (25). To propose improvements in the sensitivity of instruments to capture issues related to the quality of life, they must be specified to effectively encompass patient preferences in the context of incorporating technologies, through the integration of experience of patients in the work processes of the different HTA agencies.

Despite all the negative impacts addressed, the benefit that integrative practices provided for the cancer patients interviewed is notorious, when it comes to mental health and good prognosis. Traditionally, researchers tend to focus on the negative consequences of cancer, however going beyond this one-sided view and studying the positive aspects also promote improvements in the line of care. This finding corresponds to the process of personal development and can be called post-traumatic growth, taking into account that breast cancer is a psychosocial process and includes positive and negative consequences for the individual (26).

The present study highlighted the perspectives of patients diagnosed with breast carcinoma regarding the challenges of the disease and their preferences about the lines of treatment. Given the relevance of the presented findings and the scarcity of studies that reflect this scenario, it is necessary to explore the experiences of women in this context, to ensure improvements in the process of health technologies assessment are patient-centered, encompassing the cultural and socioeconomic aspects of the different contexts and countries.

## Data Availability

The original contributions presented in the study are included in the article/Supplementary materials, further inquiries can be directed to the corresponding author/s.
